# Supplementary material for: History of incarceration and age-related neurodegeneration: Testing models of genetic and environmental risks in a longitudinal panel study of older adults
Source: PLoS One. 2023 Dec 4;18(12):e0288303. doi: 10.1371/journal.pone.0288303 (PMC10695383; doi:10.1371/journal.pone.0288303)
Supplement: S1 Table — Lifetime incarceration predicts hazard of cognitive impairment independent of APOE-ε4 genotype and additional covariates. (DOCX) [file pone.0288303.s001.docx]

| **S1 Table**. Cox proportional hazard model of lifetime incarceration and *APOE-ε4* genotype on first cognitive impairment in the HRS (*N_Person-years_* = 117,142; *N_Cases_* = 10,031) | | | | | | | | | | | | |
| --- | --- | --- | --- | --- | --- | --- | --- | --- | --- | --- | --- | --- |
|  | Model S1.1 | | Model S1.2 | | Model S1.3 | | Model S1.4 | | Model S1.5 | | Model S1.6 | |
|  | (baseline adjustment) | | (baseline adjustment) | | (baseline adjustment) | | (full adjustment) | | (baseline adjustment) | | (full adjustment) | |
| Variable1 | **HR^2,3^** | **95% CI^3^** | **HR^2,3^** | **95% CI^3^** | **HR^2,3^** | **95% CI^3^** | **HR^2,3^** | **95% CI^3^** | **HR^2,3^** | **95% CI^3^** | **HR^2,3^** | **95% CI^3^** |
| APOE-*e4* allele count |  |  |  |  |  |  |  |  |  |  |  |  |
| One copy | 1.25*** | [1.16, 1.35] |  |  | 1.25*** | [1.16, 1.35] | 1.25*** | [1.16, 1.35] | 1.27*** | [1.17, 1.38] | 1.27*** | [1.18, 1.38] |
| Two copies | 1.68*** | [1.35, 2.09] |  |  | 1.70*** | [1.37, 2.11] | 1.67*** | [1.34, 2.08] | 1.77*** | [1.42, 2.22] | 1.75*** | [1.40, 2.19] |
|  |  |  |  |  |  |  |  |  |  |  |  |  |
| Lifetime Incarceration |  |  | 1.40*** | [1.25, 1.58] | 1.40*** | [1.25, 1.57] | 1.27*** | [1.13, 1.43] | 1.49*** | [1.30, 1.71] | 1.35*** | [1.18, 1.55] |
|  |  |  |  |  |  |  |  |  |  |  |  |  |
| Lifetime Incarceration |  |  |  |  |  |  |  |  |  |  |  |  |
| × One copy |  |  |  |  |  |  |  |  | 0.83 | [0.65, 1.07] | 0.84 | [0.66, 1.08] |
| × Two copies |  |  |  |  |  |  |  |  | 0.53 | [0.19, 1.46] | 0.49 | [0.18, 1.36] |
| ^1^The “baseline” adjustment for all models included sex, race/ethnicity, high school completion, and HRS cohort. The “full” adjustment (models S1.4, S1.6) also adjusted for stroke status, alcohol intake, BMI, depression symptoms, diabetes status, hearing difficulty, hypertension, household income, (light) physical activity level, smoking history, social isolation, childhood financial hardship, and childhood traumatic brain injury. | | | | | | | | | | | | |
| ^2^*p<0.05; **p<0.01; ***p<0.001 | | | | | | | | | | | | |
| ^3^HR = Hazard Ratio, CI = Confidence Interval | | | | | | | | | | | | |
