# Supplementary material for: History of incarceration and age-related neurodegeneration: Testing models of genetic and environmental risks in a longitudinal panel study of older adults
Source: PLoS One. 2023 Dec 4;18(12):e0288303. doi: 10.1371/journal.pone.0288303 (PMC10695383; doi:10.1371/journal.pone.0288303)
Supplement: S2 Table — Lifetime incarceration predicts risk of cognitive impairment in a dose-response pattern. (DOCX) [file pone.0288303.s002.docx]

| **S2 Table**. Mixed effect Poisson regression of cognitive status on *APOE-ε4* genotype and lifetime incarceration duration in the HRS (*N_Observation_* = 73,511; *N_Cases_* = 11,268) | | | | | |
| --- | --- | --- | --- | --- | --- |
|  | Model S2.1 | |  | Model S2.2 | |
|  | (baseline adjustment) | |  | (full adjustment) | |
| Variable*^1^* | **IRR*^2,3^*** | **95% CI*^3^*** |  | **IRR*^2,3^*** | **95% CI*^3^*** |
| *APOE- ε4* allele count |  |  |  |  |  |
| One copy | 1.24*** | [1.15, 1.33] |  | 1.23*** | [1.15, 1.32] |
| Two copies | 1.58*** | [1.27, 1.97] |  | 1.54*** | [1.25, 1.89] |
|  |  |  |  |  |  |
| Lifetime incarceration duration |  |  |  |  |  |
| Less than one month | 1.36*** | [1.18, 1.56] |  | 1.22** | [1.08, 1.39] |
| One month or more | 1.61*** | [1.35, 1.93] |  | 1.32** | [1.11, 1.56] |
|  |  |  |  |  |  |
| (Intercept) | 0.04*** | [0.03, 0.04] |  | 0.03*** | [0.03, 0.04] |
| ^1^The “baseline” adjustment for all models included age, sex, race/ethnicity, high school completion, and HRS cohort. The “full” adjustment (models 2.4, 2.6) also adjusted for stroke status, alcohol intake, BMI, depression symptoms, diabetes status, hearing difficulty, hypertension, household income, (light) physical activity level, smoking history, social isolation, childhood financial hardship, and childhood traumatic brain injury. | | | | | |
| ^2^*p<0.05; **p<0.01; ***p<0.001 | | | | | |
| ^3^ IRR = Incidence Rate Ratio, CI = Confidence Interval | | | | | |
