# Supplementary material for: History of incarceration and age-related neurodegeneration: Testing models of genetic and environmental risks in a longitudinal panel study of older adults
Source: PLoS One. 2023 Dec 4;18(12):e0288303. doi: 10.1371/journal.pone.0288303 (PMC10695383; doi:10.1371/journal.pone.0288303)
Supplement: S3 Table — Lifetime incarceration predicts hazard of cognitive impairment in a dose-response pattern. (DOCX) [file pone.0288303.s003.docx]

| **S3 Table**. Cox proportional hazard model first cognitive impairment on lifetime incarceration duration and *APOE-ε4* genotype on in the HRS (*N_Person-years_* = 117,142; *N_Cases_* = 10,031). | | | | | |
| --- | --- | --- | --- | --- | --- |
|  | Model S3.1 | |  | Model S3.2 | |
|  | (baseline adjustment) | |  | (full adjustment) | |
| Variable*^1^* | **HR*^2,3^*** | **95% CI*^3^*** |  | **HR*^2,3^*** | **95% CI*^3^*** |
| *APOE- ε4* allele count |  |  |  |  |  |
| One copy | 1.25*** | [1.16, 1.35] |  | 1.25*** | [1.16, 1.35] |
| Two copies | 1.70*** | [1.37, 2.12] |  | 1.67*** | [1.34, 2.08] |
|  |  |  |  |  |  |
| Lifetime incarceration duration |  |  |  |  |  |
| Less than one month | 1.30*** | [1.13, 1.50] |  | 1.22** | [1.06, 1.41] |
| One month or more | 1.61*** | [1.34, 1.92] |  | 1.37*** | [1.14, 1.64] |
| ^1^ The “baseline” adjustment for all models included sex, race/ethnicity, high school completion, and stratified by HRS cohort. The “full” adjustment (models 2.4, 2.6) also adjusted for stroke status, alcohol intake, BMI, depression symptoms, diabetes status, hearing difficulty, hypertension, household income, (light) physical activity level, smoking history, social isolation, childhood financial hardship, and childhood traumatic brain injury. | | | | | |
| ^2^ *p<0.05; **p<0.01; ***p<0.001 | | | | | |
| ^3^ HR = Hazard Ratio, CI = Confidence Interval | | | | | |
