# Supplementary material for: History of incarceration and age-related neurodegeneration: Testing models of genetic and environmental risks in a longitudinal panel study of older adults
Source: PLoS One. 2023 Dec 4;18(12):e0288303. doi: 10.1371/journal.pone.0288303 (PMC10695383; doi:10.1371/journal.pone.0288303)
Supplement: S4 Table — APOE-ε4 genotype and Lifetime incarceration predicts risk of cognitive impairment differentially across sex, but no differences were detected across race (Black vs. White) or educational attainment (high school completion). (DOCX) [file pone.0288303.s004.docx]

| **S4 Table**. Cox proportional hazard model of *APOE-ε4* genotype and lifetime incarceration on first cognitive impairment in the HRS with interactions | | | | | | | | | | |
| --- | --- | --- | --- | --- | --- | --- | --- | --- | --- | --- |
|  | Model S4.1^4^ | | Model S4.2^4^ | | Model S4.3 | | Model S4.4 | | Model S4.5 | |
|  | (baseline adjustment) | | (baseline adjustment) | | (baseline adjustment) | | (baseline adjustment) | | (baseline adjustment) | |
| Variable*^1,3^* | **HR*^2,3^*** | **95% CI*^3^*** | **HR*^2,3^*** | **95% CI*^3^*** | **HR*^2,3^*** | **95% CI*^3^*** | **HR*^2,3^*** | **95% CI*^3^*** | **HR*^2,3^*** | **95% CI*^3^*** |
| *APOE-ε4* |  |  |  |  |  |  |  |  |  |  |
| One copy | 1.28*** | [1.18, 1.39] |  |  | 1.28*** | [1.18, 1.39] | 1.35*** | [1.23, 1.49] | 1.30*** | [1.19, 1.41] |
| Two copies | 1.68*** | [1.34, 2.12] |  |  | 1.68*** | [1.34, 2.12] | 1.75*** | [1.32, 2.33] | 1.75*** | [1.37, 2.25] |
| Lifetime incarceration | 1.45*** | [1.27, 1.64] |  |  | 1.45*** | [1.27, 1.64] | 1.73*** | [1.41, 2.13] | 1.50*** | [1.30, 1.73] |
| Race/ethnicity (Black) | 2.08*** | [1.90, 2.27] |  |  |  |  |  |  |  |  |
| Sex (male) |  |  |  |  | 0.98 | [0.91, 1.06] | 1.03 | [0.95, 1.11] |  |  |
| High school completion (no) |  |  |  |  | 1.95*** | [1.79, 2.12] |  |  | 2.01*** | [1.84, 2.21] |
|  |  |  |  |  |  |  |  |  |  |  |
| Race/ethnicity (Black) |  |  |  |  |  |  |  |  |  |  |
| × Lifetime Incarceration |  |  | 0.81 | [0.63, 1.04] |  |  |  |  |  |  |
| × One copy |  |  | 0.88 | [0.73, 1.06] |  |  |  |  |  |  |
| × Two copies |  |  | 0.7 | [0.43, 1.17] |  |  |  |  |  |  |
|  |  |  |  |  |  |  |  |  |  |  |
| Sex (male) |  |  |  |  |  |  |  |  |  |  |
| × Lifetime Incarceration |  |  |  |  |  |  | 0.75* | [0.59, 0.96] |  |  |
| × One copy |  |  |  |  |  |  | 0.82* | [0.71, 0.96] |  |  |
| × Two copies |  |  |  |  |  |  | 0.93 | [0.60, 1.45] |  |  |
|  |  |  |  |  |  |  |  |  |  |  |
| High school completion (no) |  |  |  |  |  |  |  |  |  |  |
| × Lifetime Incarceration |  |  |  |  |  |  |  |  | 0.83 | [0.66, 1.05] |
| × One copy |  |  |  |  |  |  |  |  | 0.86 | [0.73, 1.02] |
| × Two copies |  |  |  |  |  |  |  |  | 0.87 | [0.52, 1.47] |
| *N_Person-years_* | 104,582 | | 104,582 | | 117,142 | | 117,142 | | 117,142 | |
| *N_Cases_* | 8,829 | | 8,829 | | 10,031 | | 10,031 | | 10,031 | |
| ^1^ The “baseline” adjustment for all models included sex, race/ethnicity, high school completion, and stratified by HRS cohort. | | | | | | | | | | |
| ^2^ *p<0.05; **p<0.01; ***p<0.001 | | | | | | | | | | |
| ^3^ HR = Hazard Ratio, CI = Confidence Interval | | | | | | | | | | |
| ^4^ Due to a limited number of unique cases (<20) within categories of race/ethnicity across *Lifetime incarceration* and/or *APOE-ε4 genotype*, participants who self-identified as Hispanic or “other” (*N_Cases_*=1,205) were removed. Multiplicative interaction terms test for differences between participants who identified as Black or White. | | | | | | | | | | |
