# Supplementary material for: History of incarceration and age-related neurodegeneration: Testing models of genetic and environmental risks in a longitudinal panel study of older adults
Source: PLoS One. 2023 Dec 4;18(12):e0288303. doi: 10.1371/journal.pone.0288303 (PMC10695383; doi:10.1371/journal.pone.0288303)
Supplement: S1 Fig — Panel A presents time-invariant variables (those items with fewer than N = 80 missing cases were not displayed). Childhood financial difficulty did not have any missingness among individual with valid lifetime incarceration data. Contrariwise, lifetime incarceration duration, being a follow-up question for the main question about lifetime incarceration duration, had only a single case with missing data on the main incarceration question. Panel B presents the time-varying variables by year of data collection. The social isolation index, as noted in the S1 Data, had particularly high missingness. This was due, in part, to the items contributing to index that were taken from the enhanced face-to-face interview that occurred every four years for participants. (DOCX) [file pone.0288303.s005.docx]

| **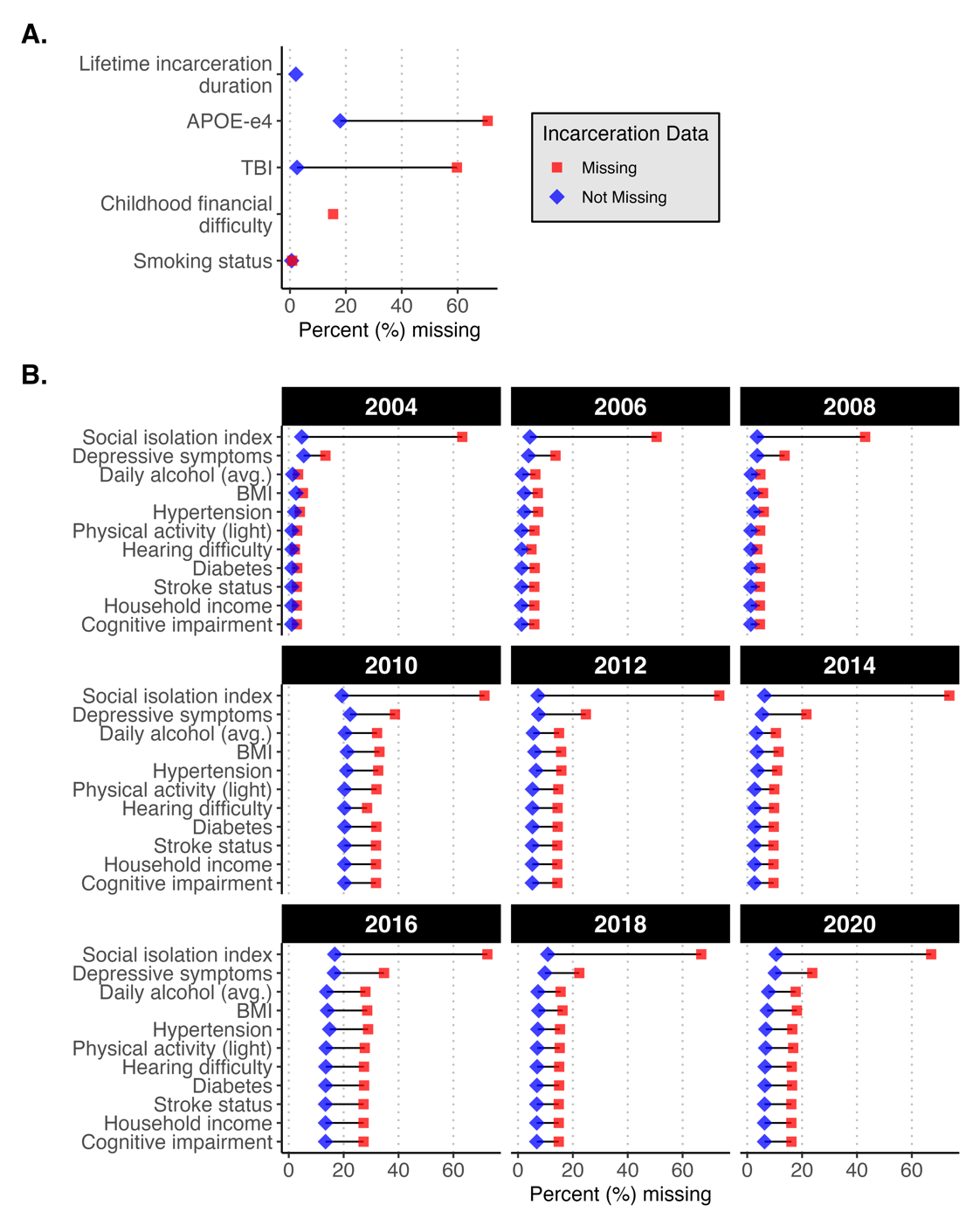** |
| --- |
| **S1 Fig. Missingness (%) across study variables in the full HRS study sample, stratified by missing status of the main lifetime incarceration question**. **Panel A** presents time-invariant variables (those items with fewer than *N*=80 missing cases were not displayed). Childhood financial difficulty did not have any missingness among individual with valid lifetime incarceration data. Contrariwise, lifetime incarceration duration, being a follow-up question for the main question about lifetime incarceration duration, had only a single case with missing data on the main incarceration question. **Panel B** presents the time-varying variables by year of data collection. The social isolation index, as noted in the **Supplemental Methods**, had particularly high missingness. This was due, in part, to the items contributing to index that were taken from the enhanced face-to-face interview that occurred every four years for participants. |
