# Supplementary material for: History of incarceration and age-related neurodegeneration: Testing models of genetic and environmental risks in a longitudinal panel study of older adults
Source: PLoS One. 2023 Dec 4;18(12):e0288303. doi: 10.1371/journal.pone.0288303 (PMC10695383; doi:10.1371/journal.pone.0288303)
Supplement: S1 File — (PDF) [file pone.0288303.s006.pdf]

HRS | HEALTH AND RETIREMENT STUDY

Sensitive Data Access Use Agreement

This Sensitive Data Access Use Agreement ("Agreement") permits the Individual User named below to obtain access to the datasets documented in *Attachment A: Sensitive Data Order Form*. A separate agreement must be completed for each User. In consideration of the HRS providing access to an HRS Sensitive Dataset to the User, the Receiving Entity, on behalf of the User, agrees:

1. To make no attempt to identify the persons in this or other HRS datasets.
2. That if the identity of any person or establishment in this file is inadvertently discovered, then (a) no use will be made of this knowledge, (b) the Director of HRS will be advised of this incident immediately, (c) the information that would identify any individual or establishment will be safeguarded or destroyed, as requested by HRS, and (d) no one else will be informed of the discovered identity.
3. To employ the following guidelines when producing tabulations for distribution:
   - Magnitude Data: Ensure that no cells/strata with n < 5 are produced.
   - Frequency Data: Apply a marginal threshold of n >= 5 and cell threshold of n >= 5 to all tabulations.
   - Protecting against complementary disclosure: Additional cells may be suppressed, i.e., complementary disclosure, to make sure the primary suppressions cannot be derived by subtraction from published marginal totals.
4. To publish only aggregate statistical summaries of the data and analyses (frequency tabulations, magnitude tabulations, means, variances, regression coefficients, and correlation coefficients), subject to the provisions above.
5. To cite HRS as the data source in any publications or research based upon these data, and to provide a copy of any publications to the HRS. The following citation should be included in any research reports, papers, or publications based on these data:

   In text: "The Health and Retirement Study data is sponsored by the National Institute on Aging (grant number U01AG009740) and is conducted by the University of Michigan."

   In references: "Health and Retirement Study. Produced and distributed by the University of Michigan with funding from the National Institute on Aging (grant number U01AG009740), Ann Arbor, MI."
6. To store and use this data set (and any data sets derived from it) in a secure computing environment.

USER *My signature certifies that I have read and acknowledge the provisions of this agreement.*

Signature/Date

Typed Name

Title

Institution

Building Address

Street Address

City, State, Zip

Phone

Email

HRS Data Download System Username

Receiving Entity Representative (if required by institution)

Signature/Date

Typed Name

Title

Institution

Building Address

Street Address

City, State, Zip

Phone

Email
