# Supplementary material for: History of incarceration and age-related neurodegeneration: Testing models of genetic and environmental risks in a longitudinal panel study of older adults
Source: PLoS One. 2023 Dec 4;18(12):e0288303. doi: 10.1371/journal.pone.0288303 (PMC10695383; doi:10.1371/journal.pone.0288303)
Supplement: S1 Data — (DOCX) [file pone.0288303.s007.docx]

**History of incarceration and age-related neurodegeneration: Testing models of genetic and environmental risks in a longitudinal panel study of older adults.**

**Supplemental methods**

**Contents:**

- Supplemental Methods
- Supplemental References

**Supplemental methods**

**Measures**

**Demographics (age, sex, race/ethnicity)**. In the HRS, age was calculated by subtracting the interview year from the record year of birth of participants. Sex was self-reported as either male or female. We combined information from two self-reported variables on race and Hispanicity to create a four-category variable for race/ethnicity (i.e., [non-Hispanic] Black, [non-Hispanic] White, Hispanic, and other).

**High school completion**. Participants reported on their completed years of education at their first wave of data collection. High school completion was created by dichotomizing self-reported years of education (≥12 years completed = “Yes”; <12 years completed = “No”).

**Stroke status**. HRS participants reported on any doctor-diagnosed stroke in the past, with any past stroke or possible stroke/transient ischemic attack/mini-strokes coded as “Yes” and “No” otherwise. For participants who were deceased or otherwise unavailable for direct interview, stroke status was obtained from proxy respondents (i.e., usually spouses). Self-reported stroke status in the HRS has previously been reported to correspond well with strokes coded according to the *International Classification of Diseases* in the Centers for Medicare and Medicaid Services records, with 74% sensitivity and 93% specificity. [1] Additionally, associations between known risk factors and incidence of stroke were similar to those reported in a sample with clinically verified stroke status. [2]

**Alcohol intake**. HRS participants were asked to report on their alcohol consumption in terms of both frequency (i.e., average number of days per week alcohol was consumed in the last three months) and quantity (i.e., average number of drinks consumed on drinking days). We created a measure of average daily alcohol intake by multiplying the frequency and quantity values together and dividing the product by seven.

**Body mass index (BMI)**. Height and weight were used to calculate BMI (kilograms/meters^2^). We used self-reported information on height (asked at baseline interview and carried forward) and weight (repeated each wave). However, if physical measurements from enhanced face-to-face interviews (administered every four years for an alternating random half-sample of the HRS) were available, we used those values.

**Depressive symptoms**. Depressive symptoms were assessed using a eight-item version of the Center for Epidemiologic Studies Depression (CESD). Participants were asked to recall if they felt any of the following feelings all or most of the time in the past week: everything is an effort, sleep was restless, felt happy (reverse coded), felt lonely, enjoyed life (reverse coded), felt sad, could not get going, had a lot of energy. Summing all eight items (coded as “Yes”=1, “No”=0) produced an index of depressive symptoms ranging from 0-8. This scale was trichotomized (i.e., 0, 1-2, and 3+ symptoms) due to limited case counts at the extreme end of the symptom range.

**Diabetes status**. HRS participants were asked if a doctor had ever diagnosed them with diabetes or high blood sugar (Yes/No).

**Hearing difficulty**. HRS participants were asked to self-rate their hearing on a five-point Likert scale (i.e., Excellent, Very Good, Good, Fair, Poor). To avoid sparse response categories, we dichotomized the original item so that responses of “Poor” or “Fair” were recoded as “impaired”, with all other responses recoded as “normal”.

**Hypertension**. HRS participants were asked if a doctor had ever told them that they had high blood pression or hypertension (Yes/No).

**Household income**. Total household income for HRS participants was calculated as the sum of earnings, pensions/annuities, social security, unemployment/workers compensation, other government transfers, household capital income, and other income for respondents and spouses. The resulting variable for household income was log-transformed in all analyses (after adding a positive constant of 1) to correct for a positive skew.

**Physical activity (light)**. HRS participants were asked to report on how frequently they engaged in sports or activities that are mildly energetic (e.g., vacuuming, laundry, home repairs). Responses were given on a five-point Likert scale (i.e., Every day, >1 day/week, 1 day/week, 1-3 days a month, Never).

**Smoking history**. HRS participants reported on their past/current use of cigarettes. We relied on the first wave of data for each participant to create a variable capturing smoking status (Ever/current smoker=1, Never smoker=0) at baseline.

**Childhood financial hardship**. Using retrospective reports from HRS participants, we created an index that summed the number of financial difficulties experienced (father was unemployed, family moved due to financial difficulties, family needed help financially, family financial status relative to other families). Each other these items was dichotomous (“Yes”=1, “No”=0) except for the item asking about relative status that was given on a three-point Likert scale (pretty well off, about average, poor). This item was dichotomized (“Poor”=1, and 0 otherwise) and all four items were summed to create an index ranging from 0-4 with high scores representing more childhood financial difficulties experienced.

**Childhood traumatic brain injury**. HRS participants were asked if (before the age of 16) if they ever experienced a head injury that was severe enough to require medical attention or caused loss of consciousness/memory (“Yes”=1, “No”=0).

**Social isolation**. Following prior research [3], we measured social isolation as an index of six items based on whether they were unmarried, lived alone, had less than monthly contact with children, other family members, or friends, and did not participate in monthly social activities (e.g., sport, social, or other club). Items regarding marriage status and living arrangements were dichotomous (“Yes”=1, “No”=0), responses to items regarding visits with children/friends/other family were given on a six-point Likert scale (three or more times a week, once a week, 2 or 3 times a month, about once a month, less than once a month, never), and responses to the item regarding social activities were given on a seven-point Likert scale (Daily, Several times a week, Once a week, Several times a month, At least once a month, Not in the past month, Never). These latter items were dichotomized so that participating in these activities less than monthly received a score of 1 and 0 otherwise. We summed these items into an index ranging from 0-6, with high scores denoting more isolation. For participants with missing data on three or fewer items, we created prorated scores by taking the average of all non-missing items and multiplying by the maximum possible number of items in the index (six).

**Note on social isolation**

Four of the six social isolation items (i.e., dealing with frequency of social activities/visits) came from the enhanced face-to-face interview that took place every four years for an alternating random half-sample of the participants. As the HRS conducts data collection biennially, this means that half of the HRS participants were offered the enhanced interview in, for example, 2006 and again in 2010, while the other half was offered the enhanced interviews in 2008 and 2012. Due to this, our measure of social isolation had a high level of missingness at the observation level—roughly 40% of observations (with otherwise complete data) lacked social isolation scores (the number of unique cases was unaffected by listwise deletion). To avoid the loss of observations, we imputed social isolation scores using two methods. First, we imputed any missing values between two valid observations (due to alternating enhanced interviews) using linear interpolation. Second, we used constant extrapolation (using the nearest valid social isolation score) to impute missing values that proceeded/followed the earliest/latest enhanced interview observations, respectively. Constant extrapolation was only extended to events with valid data on the two items that did not originate from the enhanced interview.

These steps were taken to strike a balance between (1) allowing our extended panel of covariates to more completely match the panel of modifiable risk factors identified by the 2020 report from the Lancet’s commission on dementia prevention, intervention, and care and (2) retaining the statical power needed for more precise estimates.

**Supplemental References**

1. Gilsanz, P., et al., *Changes in depressive symptoms and incidence of first stroke among middle‐aged and older US adults.* Journal of the American Heart Association, 2015. **4**(5): p. e001923.

2. Glymour, M.M. and M. Avendano, *Can self-reported strokes be used to study stroke incidence and risk factors? Evidence from the Health and Retirement Study.* Stroke, 2009. **40**(3): p. 873-879.

3. Crowe, C.L., et al., *Associations of loneliness and social isolation with health span and life span in the US Health and Retirement Study.* The Journals of Gerontology: Series A, 2021. **76**(11): p. 1997-2006.
