# Supplementary material for: History of incarceration and age-related neurodegeneration: Testing models of genetic and environmental risks in a longitudinal panel study of older adults
Source: PLoS One. 2023 Dec 4;18(12):e0288303. doi: 10.1371/journal.pone.0288303 (PMC10695383; doi:10.1371/journal.pone.0288303)
Supplement: Attachment — Submitted filename: Responses to reviewers.docx [file pone.0288303.s008.docx]

**Author response to reviews of:**

Manuscript PONE-D-23-19488

History of incarceration and age-related neurodegeneration: Testing models of genetic and environmental risks in a longitudinal panel study of older adults.

submitted to *PLOS ONE*

Dear Editor, Dr. Knittle:

We are pleased to resubmit our manuscript, “History of incarceration and age-related neurodegeneration: Testing models of genetic and environmental risks in a longitudinal panel study of older adults” (PONE-D-23-19488) for possible publication in the special issue of *PLOS ONE*, which focuses on aging in human health and disease. We again note that our study has not been published before nor is it currently under review at any other journal.

As you will see, we have taken the Reviewers’ comments seriously and made significant changes to the manuscript based on their suggestions. We focused heavily on expanding our analysis in two key ways: (1) expanding the panel of covariates included in our main analysis to cover those modifiable environmental risk factors identified in the 2020 report from the Lancet’s commission on dementia prevention, intervention, and care; and (2) including additional subgroup analyses where we explore the heterogeneity of our results across the demographic dimensions of sex, race/ethnicity, and educational attainment. We also improved the introduction and discussion sections by attempting to better integrate them with the literature on the social determinants of health literature. Lastly, as noted by the Editor and Reviewers we have removed all instances of terminology that may be perceived as derogatory (e.g., inmate, elderly) in favor of person-centered language. We hope this latter change will better facilitate the dissemination of our findings on the important topic of health disparities among incarcerated persons.

We sincerely appreciate the Reviewers’ time and effort. Their feedback has greatly improved the overall quality of the manuscript. We wish to note that a coding error present in the original analysis caused many of the oldest individuals in the sample to be dropped from the analysis (as evidenced by the absence of the original HRS birth cohort in our Table 1). This error has been rectified. The results did not change, although our analytic sample size increased. Should follow-up questions exist, we look forward to responding.

In our summary of revisions, each of the Reviewers’ comments appears in bold print followed immediately by our response. Major additions or revisions to the text have been highlighted in yellow throughout the manuscript.

We are thankful for your consideration and look forward to your decision.

Sincerely,

Authors

**REVIEWER 1**

**RC 1.1.	My review was very favorable and recommend acceptance with minor edits. This was an interesting and important study, that utilized data from the Health and Retirement Study, to examine the separate and combined effects of history of incarceration and APOE-ε4 genotype on cognition. The study was well -designed and the manuscript was clearly written, however some considerations for the authors are listed below.**

AR.	We appreciate the positive response of Reviewer 1, as well as the considerations they raise. We have attended to each point below.

**RC 1.2.	Provide reference for TICS (Line 120-125).**

AR.	We have added the appropriate, original reference for the TICS measure of cognitive function in the section on measures in the Methods.

- Brandt J, Spencer M, Folstein M. The telephone interview for cognitive status. Neuropsychiatry Neuropsychology and Behavioral Neurology. 1988;1(2):111-7.

**RC 1.3.	What is the reliability for self-report of stroke? Provide references.**

AR.	We thank Reviewer 1 for raising this issue. The validity and reliability of self-reported stroke is an important topic in population health research that has been specifically studied in the HRS context. In the new section on measurement in the **Supplemental Materials**, we make note of past efforts to validate the self-reported stroke measure in the HRS using external clinical samples. The overall conclusion is that the HRS self-report approach is generally valid and consistent with clinical assessments of stroke status.

**“Stroke status**. HRS participants reported on any doctor-diagnosed stroke in the past, with any past stroke or possible stroke/transient ischemic attack/mini-strokes coded as “Yes”, and “No” otherwise. For participants who were deceased or otherwise unavailable for direct interview, stroke status was obtained from proxy respondents (i.e., usually spouses). Self-reported stroke status in the HRS has previously been reported to correspond well with strokes coded according to the *International Classification of Diseases* in the Centers for Medicare and Medicaid Services records, with 74% sensitivity and 93% specificity. [1] Additionally, associations between known risk factors and incidence of stroke were similar in the HRS to those reported in a sample with clinically verified stroke status.[2]”

**RC 1.4.	In Table 1, provide definitions for HRS cohort in footnotes.**

AR.	We thank the Reviewer 1 for raising this lack of clarity. We have updated **Table 1** according to comments addressed below and have taken the opportunity to clarify to what is meant by “HRS cohort”. Specifically, we changed the label to “HRS cohort (birth year)” to indicate that cohorts are defined by a range of birth years. We have also listed full cohort names in the note at the bottom of the table.

**RC 1.5.	Were sex- and race-stratified analyses conducted? If not, I would explore differences by race and sex since incarcerated history differed significantly by race and sex, and the relationships between cognition and incarceration may also differ according to these factors.**

AR.	We thank Reviewer 1 for noting this point on important follow-up analyses (a point also taken up by other reviewers). We have added a new section to our analytic plan and results sections wherein we undertake the kind of subgroup analysis suggested by the reviewer (although we additionally explored the demographic characteristics of early educational attainment due to its relevance for cognitive outcomes later in life). The following excerpt describes the approach taken:

“Next, we performed two sets of subgroup analyses to better contextualize results. First… Second, we included interaction terms between our focal predictors and the variables of sex, race/ethnicity, and high school completion. This analysis allowed us to probe our results for heterogeneity across key demographic dimensions (**Table 3**). Note: as both subgroup analyses involved potential interaction terms between categorical variables, we ensured adequate statistical power and reduced the risk of violating the anonymity of participants by establishing a minimum case count of twenty unique cases for all subgroupings of crossed variables. Based on this criterion, we did not fit any three-way interactions (e.g., *APOE-ε4×Lifetime incarceration×Race/ethnicity*) or any interactions involving *Lifetime incarceration duration*. We also observed that some categories of race/ethnicity did not meet this criterion in our sample. Rather than foregoing this analysis, we removed individuals categorized as either Hispanic or “other” and estimated a separate model comparing Black/White participants across our focal predictors (e.g., *Race×APOE-ε4, Race×Lifetime incarceration*).”

Results of the above subgroup analysis are reported in **Table 3**.

**RC 1.6.	Are any data available on substance use and substance use history? Persons with substance use may be more likely to be incarcerated and also develop cognitive impairment and this should be accounted for.**

AR.	Again, we thank Reviewer 1 for identifying an important area of improvement for our analysis. We have taken the reviewers suggestion to expand the list of covariates we consider. In our main analyses (reported in **Tables 2** & **S1**), we now include two sets of model covariates: a “baseline” adjustment (age, sex, race/ethnicity, high school completion, and HRS cohort) and a “full” adjustment (including many additional covariates for risk factors including alcohol consumption). For more details, see our response to **RC 2.3** below.

**RC 1.7.	As noted, the absence of data on time spent incarcerated, number of incarceration events, and most recent incarceration is a significant limitation.**

AR.	We agree that the absence of more nuanced and precise measurement of the incarceration experience/history of HRS participants is a significant limitation of the data. As noted by another Reviewer, however, there was an additional dimension across which we might examine the incarceration experience: lifetime incarceration duration. We now include an examination of this aspect of incarceration history in the first of the aforementioned subgroup analyses (see our response to **RC 1.5**).

“Next, we performed two sets of subgroup analyses to better contextualize results. First, we decomposed our measure of *Lifetime incarceration* into two categories of total lifetime incarceration duration (i.e., less than one month; more than one month) and re-estimated our main models. This effectively tests for a possible dose-response relationship between lifetime incarceration and cognitive impairment.”

The results of this subgroup analysis are reported in the “Subgroup analyses” section of the Results, as well as **Tables S2-3**.

**REVIEWER 2**

**RC 2.1	Authors address an important public health issue that is only recently gaining more attention in the literature – the potential association between incarceration and cognitive impairment. The authors sought to determine if incarceration and APOE-E4 genotype have independent or multiplicative associations with cognitive impairment as identified via the TICS. They used Health and Retirement Study data as HRS is one of few longitudinal datasets of older individuals that includes information on incarceration history.**

AR.	We thank Reviewer 2 for their time and effort in providing very thorough and helpful feedback. We have addressed all the concerns Reviewer 2 raised below.

**RC 2.2	Throughout the manuscript, the authors use terms such as inmates and offenders. There is a push to get away from this type of derogatory language and use more person-centered/humaninzing language such as “persons who have been incarcerated” or “persons convicted of a crime.” Relatedly, the authors also use the term elderly in the manuscript. This term should be replaced with language such as “older persons” or “older people.” Moreover, because the study includes those age 50 and older, the authors may consider referring to participants as those in mid to late life.**

AR.	We would like to thank Reviewer 2 for bringing this issue to our attention. We agree that person-centered language is preferred for its humanizing qualities. We have thus removed all language that might be considered de-humanizing/derogatory and replaced those passages with person-centered terminology, both in terms of people who have experienced incarceration and people who are older.

**RC 2.3.	It is unclear from the supplementary figure how many individuals were missing information on incarceration? It would be helpful to know if those who are missing data on incarceration are also more likely to be missing data on cognition and genetic factors.**

AR.	We agree with Reviewer 2 and have worked to improve the clarity of the supplementary figure they mention. We have re-plotted the data on variable missingness but stratified the data by missingness on lifetime incarceration. This adjustment now allows for better assess how the HRS participants with/without valid data on lifetime incarceration differ with regard to the missingness across the other study variables.

**RC 2.4.	On page 5 (lines 88-90), the authors mention 12 modifiable factors that have been linked to dementia. Many of these variables are included in the HRS data (e.g., depression, alcohol, physical inactivity, diabetes, hypertension). These variables have also been found to be associated with incarceration. It is unclear why the authors mention this important potential confounders, yet they do not control for these in the adjusted models, particularly when they control for variables such as stroke and social origins index? The authors should provide some information regarding why certain variables were or were not selected to be included in the adjusted models. It is also highly recommended that the authors repeat the analyses controlling for these additional variables as time-varying covariates.**

AR.	We thank Reviewer 2 for raising this point. The HRS is a rich dataset and does indeed provide information on nearly all the aforementioned modifiable risk factors for neurodegeneration (excepting only air pollution—though external linkages have been made by independent researchers, data on air pollution are nor provided by the HRS). As the reviewer notes, however, many of these risk factors are also associated with incarceration. This presented us with a quandary: because the HRS (1) does not include information on the timing of incarceration and (2) only begins data collection in mid-adulthood (i.e., well after the peak ages for incarceration risk) we did not have the means to determine if the risk factors associated with neurodegeneration acted as confounders, mediators, or both. We originally opted for the more conservative strategy of adjusting for only a minimal number of covariates with the following qualities: (1) coverage of the major demographic dimensions, ensuring the model was properly specified and interpretable (e.g., age, race/ethnicity, sex); (2) the temporal order of covariates and incarceration could be safely assumed (e.g., childhood financial difficulty likely occurred prior to any incarceration experience); or (3) were likely too impactful for the present analysis to ignore (e.g., stroke status is highly pertinent for cognitive function tests in sample of older people). This strategy, though imperfect, attempted to avoid the issue of statistical overcontrol, when mediators are treated as confounder thereby introducing bias to the model estimates.

We recognize that this strategy was not wholly satisfactory, so we have adopted a more comprehensive, two-step approach to incorporating Reviewer 2’s suggestions. We now include two types of models in our main results (**Tables 2** and **S1**): models with a “baseline” adjustment (i.e., age, sex, race/ethnicity, high school completion, and HRS cohort) and models with a “full” adjustment (adding the following covariates to the “baseline” model: hypertension, hearing difficulty, smoking status, obesity, depression, physical (in)activity, diabetes, social isolation, alcohol consumption, and traumatic brain injury—we also add childhood financial difficulty, household income, and stroke status). We draw attention to our choice of variables in the Methods section as being a very close approximation of those risk factors identified in the 2020 report from the Lancet’s commission on dementia prevention, intervention, and care:

*“Covariates*. Every model included a “baseline” adjustment for age, self-reported sex, self-reported race/ethnicity, high school completion (Yes/No), and HRS birth cohort as covariates. We also estimated models with “full” adjustment using an extended panel of covariates that was designed to capture the leading modifiable environmental (non-genetic) risk factors for neurodegenerative diseases as outlined in the 2020 report from the Lancet’s commission on dementia prevention, intervention, and care. This panel of environmental risk factors included: hypertension, hearing difficulty, smoking status, obesity, depression, physical (in)activity, diabetes, social isolation, alcohol consumption, and traumatic brain injury (note: air pollution exposure, one of the risk factors identified in the 2020 commission report, was not provided by HRS). To this panel, we also added childhood financial difficulty and household include to account for previous and contemporaneous differences in socioeconomic position, as well as stroke status. Full details regarding the measurement of covariates in the HRS are provided in the **Supplemental Material**.”

Immediately following this passage, we also include a section outlining our concerns around statistical overcontrol and stating that we will be focusing our interpretation on models with the “baseline” adjustment.

“Note: it is possible that many of the risk factors included in our extended panel of covariates behave as mediators and/or confounders. For instance, problematic alcohol use could behave as both a confounder (incarceration 🡨 alcohol 🡪 cognitive impairment), a mediator (incarceration 🡪 alcohol 🡪 cognitive impairment), or both (alcohol 🡪 incarceration 🡪 alcohol 🡪 cognitive impairment). As the measurement of lifetime incarceration in the HRS restricts our ability to establish temporal order among covariates and exposure to the incarceration experience, our use of the extended panel of covariates is considered exploratory and we likewise restrict our primary focus to the models with the “baseline” adjustment where temporal order with incarceration experience is more certain and statistical overcontrol (i.e., controlling for mediators) is less likely. (43)”

We hope the above changes, which we feel has greatly improved the rigor of the analysis, will ease Reviewer 2’s concerns regarding the possible confounding of the main results by known/acknowledged risk factors.

**RC 2.5.	Related to Comment #3** [RC 2.2]**, on page 16 (lines 253-255) the authors indicate that incarceration confers the same risk for cognitive impairment as APOE-E4 count. Yet, without controlling for factors such as depression and alcohol, this finding may be overstated.**

AR.	We thank Reviewer 2 for making this point. Indeed, in the new “full” adjustment models presented in **Tables 2** and **S1**, the risk for cognitive impairment conveyed by Lifetime incarceration was reduced somewhat, with effect sizes falling between those of possessing one and two copies of the *APOE-ε4* allele. We have changed the language to reflect this shift, though we maintain that the risk is still *comparable*. For example:

“We found that *Lifetime incarceration* conferred a substantive increase in the risk of cognitive impairment (**Table 2**). HRS participants with a history of incarceration were 1.5 times as likely to be cognitively impaired than their non-incarcerated peers (IRR=1.45, 95% CI=[1.29, 1.62], P<0.001) (**Model 2.2**)–a level of risk between that of individuals with one or two *APOE-ε4* alleles (e.g., IRRs=1.24-1.57)…”

“We did, however, observe a reduction in effect size in the fully adjusted model (IRR=1.26 95% CI=[1.13, 1.40], P<0.001; **Model 2.4**).”

As with the “baseline” adjustment model (Model 2.2), the effect size for *Lifetime incarceration* (IRR=1.26) in the “full” adjustment model (Model 2.4) again fell between those for possessing one or two copies of the *APOE-ε4* allele (IRRs were 1.24 and 1.54, respectively).

**RC 2.6.	Per an article cited by the authors (Garcia-Grossman), the HRS contains information on time spent incarcerated. So, it is unclear why the authors did not control for this variable as they mention on page 20 that duration of incarceration may impact outcomes.**

AR.	Reviewer 2 again brings up an important point. While the HRS does not measure incarceration with much nuance, it does at least include a measure of lifetime incarceration duration. We did consider including this measure in our original analysis, however, as our primary aim was to estimate an interaction between *APOE-ε4* genotype and incarceration, we did not want to risk estimating interactions across subcategories of crossed variables that were too sparsely populated. As the HRS is a population-based cohort, the low prevalence of individuals with longer lifetime incarceration experiences made this issue a serious concern from both a statistical and ethical standpoint (analyzing models on sparse data greatly increases risk of inadvertent identification).

Recognizing this issue, we have attempted to incorporate the measure of lifetime incarceration duration into our analysis by including it as one of the “subgroup” analyses (see also our response to **RC 1.7**). In the “Analytic strategy” subsection of the Methods, we now describe an analysis using said measure as the first of two subgroup analyses:

“Next, we performed two sets of subgroup analyses to better contextualize results. First, we decomposed our measure of *Lifetime incarceration* into two categories of total lifetime incarceration duration (i.e., less than one month; a month or more) and re-estimated our main models. This effectively tests for a possible dose-response relationship between lifetime incarceration and cognitive impairment.”

This analysis makes use of the lifetime incarceration duration measure; however, as noted in the above passage, we use a somewhat truncated version of the original variable to avoid sparse categories. Measurement of the truncated duration measure is described in the “Measures” subsection of the Methods:

“We also created a variable that decomposed the *Lifetime incarceration* into periods of incarceration duration. For individuals who indicated that they had been incarcerated, they were asked a follow-up question regarding the lifetime duration of their time spent incarcerated with responses on a four-point Likert scale (less than one month, less than one year, between 1-5years, more than 5 years). We collapsed these categories into two durations (less than one more, a month or more) to avoid sparse response categories and assigned all participants who were never incarcerated a value of “none”. The resulting three-category variable we termed *Lifetime incarceration duration*.”

However, collapsing categories was not enough to prevent sparse categories when considering the addition of multiplicative interaction terms (an issue also raised for models requested by the reviewers involving interactions with sex and race/ethnicity). We addressed this concern and stated our attempted remedy in the final passage of the “Analytic strategy” subsection of the Methods:

“Note: as both subgroup analyses involved potential interaction terms between categorical variables, we ensured adequate statistical power and reduced the risk of violating the anonymity of participants by establishing a minimum case count of twenty unique cases for all subgroupings of crossed variables. Based on this criterion, we did fit any three-way interactions (e.g., *APOE-ε4 × Lifetime incarceration × Race/ethnicity*) or any interactions involving *Lifetime incarceration duration*. We also observed that some categories of race/ethnicity did not meet this criterion in our sample. Rather than foregoing this analysis, we removed individuals categorized as either Hispanic or “other” and estimated a separate model comparing Black/White participants across our focal predictors (e.g., *Race × APOE-ε4, Race × Lifetime incarceration*).”

**RC 2.7.	Based on the findings in Table 1, there was nearly the same distribution of APOE-E4 count in those with and without a history of incarceration. Based on the similar distribution, it does not seem surprising that an interaction term between incarceration and APOE-E4 would be non-significant. Could the authors comment further in the Discussion on the added value of evaluating this interaction?**

AR.	We do not share Reviewer 2’s unsurprise at the lack of statistical interaction between *APOE-ε4* and *Lifetime incarceration* based only on the similarity of distributions of *APOE-ε4* genotype across incarceration groups. We would note that statistical interactions are based on more than bivariate distributions. Additionally, should a difference in genotypes been observed between groups, it would have suggested the presence of gene-environment correlation (i.e., *APOE-ε4* genotype somehow influencing selection into the incarceration experience). The presence of gene-environment correlation (rGE) would constitute a violation of the assumptions of our G×E model, risking the inflation of interaction effects and increasing the false positive rate.

We thank Reviewer 2 for raising this point, however, as it provided us with a valuable opportunity to better situate our G×E models in the present study. We took advantage of this opportunity and included the following preamble to the subsection in the Results entitled “Does the incarceration experience amplify genetic risk factors for cognitive impairment in older adults?”

“We did not find evidence that *Lifetime incarceration* interacts with *APOE-ε4 genotype* to produce excess risk for cognitive impairment. Prior to formally testing for statistical interaction, we observed that *APOE-ε4 genotype* did not differ across participants with/without prior incarceration experience (**Table 1**), suggesting that *APOE-ε4 genotype* did not contribute to selection into the carceral system which is a crucial assumption of G×E models (i.e., no gene-environment correlation). (47, 48)”

**RC 2.8.	On page 17 (lines 281-284) and page 18 (lines 302-304) the authors discuss risk classification for cognitive status at the time of intake. However, for older persons who spent a considerable amount of time incarcerated, classification at the time of intake may be very different than their risk classification during the extent of their incarceration. The points made on these 2 pages should likely be discussed together. The authors should clarify how risk classification at the time of intake may or may not impact future cognitive status/services related to cognition.**

AR.	We agree with Reviewer 2 that the two paragraphs needed better integration. We have reorganized these paragraphs by focusing on the need for and potential use of general assessments of aging into the intake process (paragraph 1) and integration of genetic risk factors (paragraph 2). We make the point that assessments of age-related processes (physical and cognitive) are needed to elucidate the causal processes at work in the carceral context (i.e., how variation in the incarceration experience informs within-person change in cognitive function).

**RC 2.9.	Citation #62 is more than 20 years old. Do the authors have a more recent citation that provides evidence that incarcerated persons age 50 are similar to non-incarcerated persons age 65?**

AR.	We regret that we were unable to find a more recent citation for this figure. We have drawn attention to the age of the citation in the text as follows:

“The most recent estimates and projections, though dated, indicate that incarcerated persons over the age of 50 maintain health profiles equivalent to persons outside of prison who are 65 and older.[76]”

**RC 2.10.	It is well known that incarceration is more prevalent among minority groups in the U.S. At some point, the authors should acknowledge the vast differences in the proportion of incarceration within the different race groups in the HRS sample. Although the majority of those incarcerated were white, if you look within race categories there is a very different picture: 7.6% whites incarcerated; 18.9% blacks incarcerated; and 11.5% Hispanics incarcerated. Did the authors evaluate an interaction between race and incarceration or race APOE-E4 count?**

AR.	Following the Reviewer 2’s suggestion, we did evaluate interactions between race (and sex) and our focal predictors. See our above responses to **RC 1.5** and **2.4**.

**REVIEWER 3**

**RC 3.1.	This is an interesting article that contributes to the field's understanding of the relationship of incarceration on dementia and cognitive decline. My comments mostly involve wording, language and phrasing to help better communicate the ideas and reduce stigma.**

AR.	We thank Reviewer 3 for their kind words and have attempted to changed our use of language to improve the communication of our results and reduce stigma around the import topic of health disparities among incarcerated persons.

**RC 3.2.	INTRO - Overall, this section needs to be strengthened to better document that people who are incarcerated generally have worse health than the GP (see the Bureau of Justice Statistics health briefs for data) because they generally experience more hardships in the community (housing insecurity, exposure to violence, poverty, etc.) that put them at risk for incarceration AND poor health. These same factors then overlap with risks for dementia and incarceration exacerbates risks for dementia. I suggest incorporating the social determinants of health model (SDOH) to tie things together.**

AR.	We agree with Reviewer 3 and have taken their suggestion to rely on the social determinants of health framework to better tie together the various threads laid out in the introduction. For instance, in the third paragraph we lay out the acute and chronic health consequences of incarceration. We conclude with this passage:

“Together, these factors frame incarceration as a social determinant of health (29, 30), a non-medical factor impacting health such as health-related knowledge, attitudes, beliefs, and behaviors.(31) It has been noted that incarceration may, in fact, constitute both a cause and consequence of social determinants of health as many such factors that are exacerbated by the incarceration experience also raise the risk of initial incarceration (e.g., substance use) (32). Here, we focus on the downstream consequences of incarceration as they relate to neurological health. And while it is acknowledged that formerly incarcerated persons bear a heavier than expected burden of age-related neurodegeneration (20), it is not well-understood if/how the risks associated with the incarceration experience relates the other main source of risk for neurodegeneration: genetic risk factors.”

And again, in paragraph five, when describing the modifiable environmental risk factors identified in the 2020 report from the Lancet commission:

“Indeed, the 2020 Lancet Commission report on dementia prevention, intervention, and care identified 12 modifiable risk factors that, together, accounted for 40% of dementias world-wide.(37) These included education, hearing loss, traumatic brain injury (TBI), hypertension, alcohol intake, obesity, smoking, depression, social isolation, physical inactivity, diabetes, and air pollution. Many of these risk factors are social determinants of health (e.g., alcohol intake, smoking, social isolation) and are typically the result of more “upstream” social determinants (e.g., socioeconomic position, education, criminal-legal involvement).(31)”

We believe the adoption of the SDoH framework has indeed helped provide continuity across the topics presented in the introduction.

**RC 3.3.	Please avoid "offenders" and "inmates" where possible. Although these are still commonly used in the criminal justice system, the academic field and health field have moved away from using these terms.**

AR.	We have removed all instances of stigmatizing language noted by Reviewer 3, as well as those terms relating to older people (see our response to **RC 2.2**).

**RC 3.4.	"Most former inmates survive the high-mortality period immediately following release and eventually succumb to the same chronic conditions that drive mortality for the general population such as cancer, cardiovascular disease, and neurodegenerative diseases." This sentence needs to be rethought, while not factually incorrect, it obscures the point that people with CJ involvement generally have worse health than GP, and buries that people with a history of incarceration are likely going to die from those conditions earlier than the GP.**

AR.	We thank Reviewer 3 for pointing out this ambiguity. It was not our intension to obscure the heavy health burden of formerly incarcerated persons. We have updated the abovementioned passage, and it now reads:

“Most of the formerly incarcerated survive the high-mortality period immediately following release and eventually succumb to the same chronic conditions that drive mortality for the general population—albeit at exaggerated rates, relative to the general population (19), resulting in earlier mortality.(20-22) Examples of chronic conditions associated with past incarceration include cancer, cardiovascular disease, and neurodegenerative diseases such as Alzheimer’s Disease and Related Dementias (ADRDs).(19, 23-26)”

**RC 3.5.	“Minoritized groups” is odd phrasing. And it's not the race that is the risk factor, it's the racism they experience, so please be careful with how that is worded.**

AR.	We have removed all instances of the phrase “minoritized groups”.

**RC 3.6.	The discussion does a better job of tying ideas together, but it also could use some strengthening to better connect the ideas. Again, I think the SDOH model will be helpful framing.**

AR.	We thank Reviewer 3 for this suggestion. We did indeed find the SDoH model useful in framing the topics discussed here, particularly in the introduction.
